# Reduced expression of a rhomboid protease, EhROM1, correlates with changes in the submembrane distribution and size of the Gal/GalNAc lectin subunits in the human protozoan parasite, *Entamoeba histolytica*

**DOI:** 10.1371/journal.pone.0219870

**Published:** 2020-03-05

**Authors:** Brenda H. Welter, Heather A. Walters, Lesly A. Temesvari

**Affiliations:** 1 Department of Biological Sciences, Clemson University, Clemson, South Carolina, United States of America; 2 Eukaryotic Pathogens Innovations Center (EPIC), Clemson University, Clemson, South Carolina, United States of America; Centro de Investigacion y de Estudios Avanzados del Instituto Politecnico Nacional, MEXICO

## Abstract

*Entamoeba histolytica* is a food- and waterborne parasite that causes amebic dysentery and amoebic liver abscesses. Adhesion is one of the most important virulence functions as it facilitates motility, colonization of host, destruction of host tissue, and uptake of nutrients by the parasite. The parasite cell surface adhesin, the Gal/GalNAc lectin, facilitates parasite-host interaction by binding to galactose or *N*-acetylgalactosamine residues on host components. It is composed of heavy (Hgl), intermediate (Igl), and light (Lgl) subunits. Igl is constitutively localized to lipid rafts (cholesterol-rich membrane domains), whereas Hgl and Lgl transiently associate with rafts. When all three subunits are localized to rafts, galactose-sensitive adhesion is enhanced. Thus, submembrane location may regulate the function of this adhesion. Rhomboid proteases are a conserved family of intramembrane proteases that also participate in the regulation of parasite-host interactions. In *E*. *histolytica*, one rhomboid protease, EhROM1, cleaves Hgl as a substrate, and knockdown of its expression inhibits parasite-host interactions. Since rhomboid proteases are found within membranes, it is not surprising that lipid composition regulates their activity and enzyme-substrate binding. Given the importance of the lipid environment for both rhomboid proteases and the Gal/GalNAc lectin, we sought to gain insight into the relationship between rhomboid proteases and submembrane location of the lectin in *E*. *histolytica*. We demonstrated that EhROM1, itself, is enriched in highly buoyant triton-insoluble membranes reminiscent of rafts. Reducing rhomboid protease activity, either pharmacologically or genetically, correlated with an enrichment of Hgl and Lgl in rafts. In a mutant cell line with reduced EhROM1 expression, there was also a significant augmentation of the level of all three Gal/GalNAc subunits on the cell surface and an increase in the molecular weight of Hgl and Lgl. Overall, the study provides insight into the molecular mechanisms governing parasite-host adhesion for this pathogen.

## Introduction

*Entamoeba histolytica* is an intestinal parasite that is the causative agent of amebic dysentery and amoebic liver abscesses (reviewed in [1]). The cyst form of the pathogen is the infective stage and is found in fecally-contaminated food and water. This makes this disease prevalent in the developing world where sanitation practices are inferior. In 2015, it was estimated that 2.4 billion people still lacked access to improved sanitation facilities and 946 million people still carried out open defecation practices [2]. These substantially contribute to the risk for the transmission of *E*. *histolytica*.

After ingestion, the cyst exits the stomach and enters the small intestine, where unknown triggers cause excystation. The emerging amoeboid trophozoites travel down the digestive system until they reach the large intestine, where infection is established. In the large intestine the trophozoites feed on bacteria and host cell material by endocytosis, and divide by binary fission. Invasion of the epithelial lining of the colon can result in extra-intestinal complications of infection, including liver abscess. During colonization and invasion of the host, trophozoites adhere to numerous host-derived and host flora-derived ligands, including epithelial cells [3], red blood cells (RBCs) [4,5], extracellular matrix components (e.g., collagen [4,6] and fibronectin [7]), intestinal flora [8,9], colonic mucins [8–10], and leukocytes [11,12]. Since adhesion is one of the first steps in host colonization, and facilitates uptake of nutrients by endocytosis, it may be one of the most important virulence functions for this parasite.

A number of parasite cell surface adhesins have been discovered (reviewed in [13]); however, the best characterized is the heterotrimeric protein complex known as the galactose/N-acetylgalactosamine lectin (Gal/GalNAc lectin). This protein multimer binds to galactose and N-acetylgalactosamine residues on host ligands and is composed of heavy (Hgl), light (Lgl), and intermediate (Igl) subunits. Hgl is a transmembrane protein that possesses an exoplasmic carbohydrate binding domain and is disulfide-linked to glycophosphatidylinositol (GPI)-anchored Lgl. The Hgl-Lgl dimer non-covalently associates with Igl, which is also anchored to the membrane by GPI-linkage. Both Hgl and Igl share sequence similarity with β integrins [14–17], suggesting that they may also play a role in signaling. For example, Hgl is thought to facilitate signaling and adhesion not only by binding extracellular ligands, but also by interacting with proteins in the intracellular space of the parasite, via its cytoplasmic tail [15].

Mechanisms regulating the function of the Gal/GalNAc lectin are not well understood; however, it has been shown that lipid rafts may play a role [4,6,18,19]. Lipid rafts are membrane domains that are rich in cholesterol and sphingolipid. Signaling proteins are known to interact within these domains. It was previously shown that the majority of Igl is found in raft-like membranes, whereas the majority of Hgl and Lgl is found in non-raft membrane [18]. However, exposing *E*. *histolytica* cells to a source of cholesterol [19] or several host ligands, including RBCs [4] or collagen [6], results in enrichment of Hgl and Lgl in rafts and thus, co-compartmentalisation of all three subunits. Colocalization of the subunits in rafts is accompanied by an increase in the ability of the amoebae to adhere to host components in a galactose-specific manner [19]. Removal of cholesterol disrupts lipid rafts and inhibits the adhesion of *E*. *histolytica* trophozoites to host cells [18] and collagen [6]. Together, these data suggest that there is a correlation between submembrane location and function of the Gal/GalNAc lectin, and that lipid rafts may serve as a platform for the assembly, modification, and/or functional regulation of proteins involved in parasite-host interaction.

Cells must also possess mechanisms to modulate or dismantle adhesion junctions. Rhomboid proteases are a family of intramembrane proteases that participate in a wide variety of cellular functions including cell signaling, mitochondrial homeostasis, quorum sensing, protein translocation across membranes, and the regulation of adhesion junctions (reviewed in [20]). They are conserved from bacteria to mammals and their role in regulating parasite-host interactions (reviewed in [21]) is established in *Toxoplasma* [22–26], *Plasmodium* [27–29], *Trichomonas* [30], and *Entamoeba* [31–33].

In particular, for *E*. *histolytica*, Hgl is thought to be a substrate of the *E*. *histolytica* rhomboid protease, EhROM1, because it can be cleaved by EhROM1 when they are co-expressed in a mammalian cell system [31]. Knocking down expression of EhROM1, using an epigenetic silencing approach, results in reduced adhesion to host cells and reduced erythrophagocytosis [32]. Overexpression of a dominant negative catalytically inactive mutant of EhROM1 also causes defects in host cell binding [33]. Finally, overexpression of the catalytically inactive mutant or knocking down expression, using an RNAi-based method, gives rise to mutant cells that are less cytotoxic, hemolytic, and motile than control cells [33]. Together, these observations support the role of EhROM1 in parasite-host interactions.

Since rhomboid proteases have an intramembrane position, a logical conjecture is that lipid composition regulates activity and compartmentalization regulates enzyme-substrate contact. In support of this, the activity of both prokaryotic and eukaryotic rhomboid proteases can be influenced by membrane composition *in vitro* [34] and pharmacological perturbation of cellular membranes *in vivo* can alter the activity of at least one rhomboid protease, human RHBDL4 [35].

Given the importance of compartmentalization for both rhomboid proteases and the Gal/GalNAc lectin, we sought to gain insight into the relationship between rhomboid protease activity and submembrane location of the lectin in *E*. *histolytica*. We demonstrate that EhROM1 is localized to buoyant triton-insoluble membrane, reminiscent of rafts, and loss of rhomboid protease expression correlates with an enrichment of the Gal/GalNAc lectin at the cell surface and in lipid rafts. We also show that the molecular weights of Hgl and Igl are increased in the absence of EhROM1 activity, suggesting that they are substrates of this protease. Overall, the study provides insight into the molecular mechanisms governing parasite-host adhesion for this pathogen.

## Materials and methods

### Strains and culture conditions

The generation of an *Entamoeba histolytica* cell line with RNAi-mediated reduced expression of EhROM1 is described elsewhere [33], and was generously provided by Dr. Upinder Singh (Division of Infectious Diseases, Dept. of Internal Medicine, Dept. of Microbiology and Immunology, Stanford University School of Medicine, Stanford, CA, USA). Both mutant and wildtype *E*. *histolytica* trophozoites (strain HM-1:IMSS) were grown axenically at 37°C in TYI-S-33 media [36] in 15 ml glass screw cap tubes.

### Pharmacological inhibition of rhomboid protease activity

To inhibit rhomboid protease activity, parasites (3.5 x 10^6^ cells/ml) were treated with 100 μM 3,4-dichloroisocoumarin (DCI) (Sigma-Aldrich, St. Louis, MO). DCI was dissolved in dimethyl sulfoxide (DMSO) and applied to the parasites for 2 h at 37°C. Control parasites were treated with DMSO alone.

### RNA extraction and RT-PCR

Total RNA was purified from both mutant and wildtype cells using TRIzol (Ambion/Life Technologies Carlsbad, CA). To remove any contaminating genomic DNA, the RNA was treated with DNAseI (Promega, Madison, WI). RNA was reverse transcribed and cDNA was generated using the Superscript^™^ III First Strand Synthesis Kit (Invitrogen, Carlsbad, CA). The cDNA served as a template for PCR using primers specific for EhROM1(EHI_197460; forward: 5’GCTTCAGGTCGTTGCTTGG3’and reverse: 5’CACTGCAAGACATATAATTGGGCA3’) or primers specific for a load-control small subunit ribosomal RNA (16s-like rRNA; X61116; forward:5’AGGCGCGTAAATTACCCACTTTCG3’ and reverse 5’CACCAGACTTGCCCTCCAATTGAT3’), as previously described [32]. PCR products were analyzed by agarose gel electrophoresis. PCR reactions, in which the reverse transcriptase step was left out, or in which cDNA was left out, served as controls.

### Lipid raft isolation and characterization by western blot analysis

Isolation and characterization of lipid rafts were carried out as previously described [18]. 3 × 10^6^ *Entamoeba* cells were harvested by centrifugation (500 x g, 5 min) at 4°C and then incubated for 30 min in ice cold extraction buffer consisting of 10 mM Tris-HCl [pH 7.6]), protease inhibitors (40 mM sodium pyrophosphate, 0.4 mM dithiothreitol, 0.1 mg of phenylmethylsulfonyl fluoride/ml, 2 mM EDTA, 1 mM EGTA, 3 mM sodium azide) and 0.5% (v/v) Triton X-100. The lysate was centrifuged (14,000 × g, 5 min) at 4°C. The Triton-insoluble pellet was resuspended in 80% (wt/vol) sucrose in extraction buffer. Equal volumes of 80 (containing the pellet), 50, 30, and 10% (wt/vol) sucrose solutions, in extraction buffer, were used to generate a discontinuous sucrose gradient. The gradients were centrifuged in a Beckman Coulter (Indianapolis, IN) Optima^™^ MAX-XP ultracentrifuge (125,000 × g for 16 h at 4°C). Twenty equal volumes (140 μl/fraction) were collected from the gradients and treated with trichloroacetic acid (TCA) to precipitate the proteins [37]. Proteins were dissolved in 4X LDS buffer (Invitrogen, Carlsbad, CA) containing 2-mercaptoethanol (10% [vol/vol].

To assess the level of protein in each fraction, and to observe fraction-to-fraction shifts in the relative abundance of protein, TCA-precipitated preparations were analyzed by SDS-PAGE and western blot analysis as previously described [4,18,19]. Primary antibodies for western blot characterization included a mixture of monoclonal α-Lgl antibodies (3C2, IC8, IA9, and ID4) (1:4,000 dilution), polyclonal α-Hgl antibodies (1:5,000 dilution) [38], a mixture of monoclonal α-Igl antibodies (3G5-A3-G3, 5H1-F11-D11, and 4G2-D8-H1) (1:4,000 dilution), or polyclonal α-EhROM1 antibodies (1:50 dilution). Monoclonal antibodies recognizing Igl and Lgl were generously provided by Dr. William A. Petri, Jr. (Division of Infectious Diseases and International Health, University of Virginia, Charlottesville, VA, USA). Polyclonal antibodies recognizing EhROM1 were generously provided by Dr. Upinder Singh (Division of Infectious Diseases, Dept. of Internal Medicine, Dept. of Microbiology and Immunology, Stanford University School of Medicine, Stanford, CA, USA). Bethesda, MD). Commercial HRP-conjugated polyclonal goat anti-rabbit or goat anti-mouse secondary antibodies (MP Biomedicals, Solon, OH) were utilized at 2 μg/ml.

The membrane was processed using the Enhanced ChemiLuminescence Western blotting detection system (Thermo Scientific, Hercules, CA, USA) and exposed to X-ray film. X-ray film was developed on a Mini-Med 90 X-ray film processor (AFP Imaging, Elmsford, NY). The films were analyzed by densitometry using ImageJ software (version 1.51; U.S. National Institutes of Health). Densitometry values for each protein (Hgl, Lgl, or Igl) were summed across all fractions. The densitometry value in each fraction was then converted to a percentage of the sum. Thus, the data are presented as the percent of protein in each fraction relative to the percent of protein in every other fraction.

### Cell surface biotinylation

Control and T-EhROM1-s cells (6.0 × 10^5^) were surface biotinylated and purified by avidin affinity chromatography using the Pierce Cell Surface Protein Isolation Kit (Pierce Biotechnology, Rockford, IL, USA) according to manufacturer’s specifications. Flow-through fractions and biotinylated surface proteins, captured by avidin affinity chromatography, were resolved by SDS-PAGE and analyzed for Hgl, Lgl, Igl and actin by western blotting and densitometry as described above.

### Immunoprecipitation

Triton-insoluble membrane was isolated from wildtype and T-EhROM1-s cells as described above and fractionated on sucrose gradients. Fractions 11–12 (lipid rafts), 18–19 (actin-rich membrane) or TSS were used for immunoprecipitation assays. These narrow ranges of fractions were chosen to increase the probability that no membrane type (e.g., lipid rafts) would be contaminated with any other membrane type (e.g. actin-rich membrane). Combined fractions 11 and 12, 18 and 19 or TSS were pre-cleared by incubation with 1 X 10^7^ Dynabeads magnetic beads conjugated to sheep α-mouse IgG (Invitrogen Dynal AS, Oslo Norway) at 4°C for 2 h. The beads were collected in a microfuge tube magnetic separation stand (Promega, Madison, WI) and discarded. Protease inhibitors (as described above) and a mixture of monoclonal α-Hgl antibodies (1G7:3F4; ratio 3:1 to give a final antibody concentration of 0.01 μg/μl) were added to the pre-cleared fractions. The fractions were incubated for 1 h at room temperature with constant rotation. Monoclonal antibodies recognizing Hgl were generously provided by Dr. William A. Petri, Jr. (Division of Infectious Diseases and International Health, University of Virginia, Charlottesville, VA, USA). 1.5 X 10^7^ sheep α-mouse IgG-conjugated magnetic beads were then added to the samples, which were incubated overnight at 4°C with constant rotation. Beads were collected in the magnetic separation stand, washed 6X with PBS, and boiled for 4 min in 4X LDS buffer and 2-mercaptoethanol (10% [vol/vol] final concentration). Samples were stored at -80°C until analyzed by western blotting as described above.

### Statistical analyses

All values are given as means ± standard deviations (SD). Statistical analyses were performed using GraphPad Prism V.6.05 with a one-way analysis of variance (ANOVA) and a Tukey-Kramer multiple comparison test. *P* values of less than 0.05 were considered statistically significant. *P* values less than 0.01 were considered highly statistically significant.

## Results

### Pharmacological inhibition of rhomboid proteases alters the submembrane location of the Gal/GalNAc subunits

Intramembrane protease-substrate interactions may be regulated by spatial segregation of either the enzyme or target protein in sub-membrane domains, such as lipid rafts [39]. Furthermore, lipid composition may influence intramembrane protease activity [34,35]. Thus, a logical prediction is that intramembrane proteolysis by rhomboid proteases may regulate the submembrane distribution of other membrane proteins.

To determine if rhomboid protease activity influenced the submembrane distribution of the subunits of the Gal/GalNAc lectin, we characterized lipid rafts in trophozoites exposed to DCI, a compound which inhibits rhomboid proteases by alkylating its active-site histidine [34]. DCI has been previously used to study EhROM1 function in *E*. *histolytica* [32]. The characteristic composition of lipids in lipid rafts confers detergent resistance to these domains. Thus, the initial step in their purification was extraction with cold Triton X-100. However, the resulting detergent-resistant membrane (DRM) consists of both lipid raft and actin-rich membrane that differ in their buoyant density. Thus, these two membrane domains were further resolved by sucrose density gradient centrifugation.

**Fig 1:**
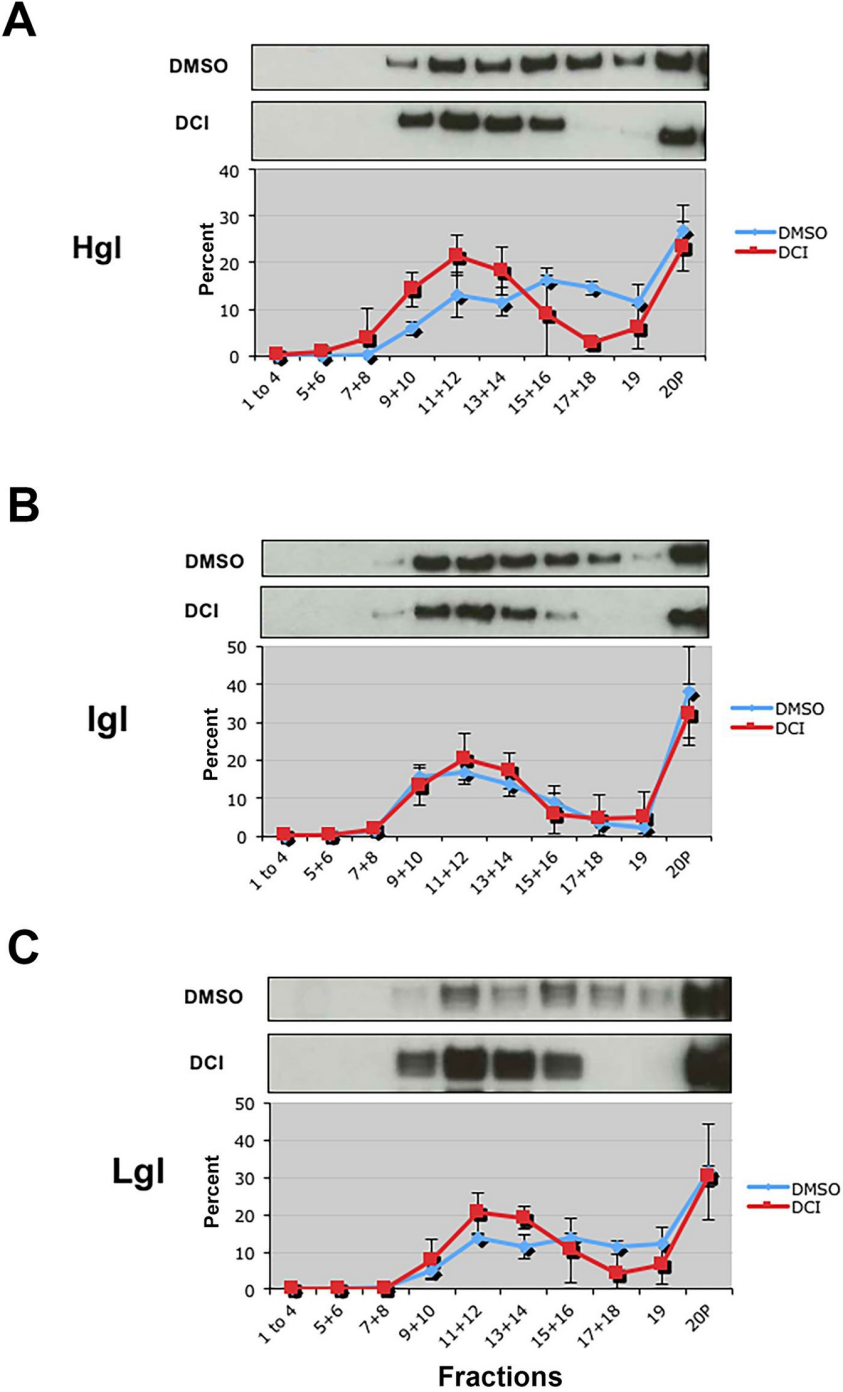
Inhibition of rhomboid protease activity with 3,4-dichloroisocoumarin (DCI) induces enrichment of the heavy (Hgl) and light (Lgl) subunits of the Gal/GalNAc lectins in lipid rafts. *E*. *histolytica* trophozoites were treated with a 100 μM DCI or diluent (DMSO) for 2 h. Triton-insoluble membranes were isolated and further separated by sucrose gradient density centrifugation. Nineteen fractions and the non-buoyant pellet (20P) were collected and subjected to western blot analyses using antibodies to the heavy subunit (Hgl), the intermediate subunit (Igl) or the light subunit (Lgl). Fractions 1 through 4, and pairs of fractions thereafter, were combined prior to analysis. For each of the subunits, the data presented are the means of scanning densitometry values (n ≥ 2) after conversion to a percentage relative to the values in all other fractions. Representative western blots are provided above each panel. In both control (DMSO) and treated cells (DCI) Igl was localized to fractions 9–14, which were previously shown to be raft-like [18]. In untreated cells (blue) Hgl and Lgl were distributed in lipid rafts (fractions 9–14) and actin-rich membrane (fractions 15–19). After DCI-treatment (red), Hgl and Lgl were enriched in low density lipid rafts.

*E*. *histolytica* is a divergent eukaryote and homologs to canonical lipid raft markers, such as the GM1 ganglioside, Thy-1, or flotillin have not been identified [40]. We previously used sucrose gradient centrifugation to show that Igl is the only protein that is constitutively localized to buoyant, cholesterol-rich, triton-insoluble membrane fractions, that are reminiscent of lipid rafts [4,18,19]. Thus, Igl serves as a marker for raft-like membranes in *E*. *histolytica*. Western blotting revealed that the raft marker, Igl, was enriched in low-density fractions (fractions 9 to 14) (Fig 1B). The enrichment of Igl in these fractions is consistent with previous reports [4,18,19]. In DMSO (diluent)-treated control cells, Hgl and Lgl were distributed in less buoyant, actin-rich fractions (fractions 17 to 19) and lipid rafts (fractions 9 to 14) (Fig 1A and 1C). On the other hand, after treatment with DCI, there was an increase in the relative level of Hgl and Lgl in lipid raft fractions (fractions 9 to 14) (Fig 1A and 1C) and a concomitant loss of these subunits in the highest density actin-rich fractions (fractions 17–19) (Fig 1A and 1C). Treatment with DCI had no impact on the submembrane distribution of Igl as it remained unchanged in the presence of the reagent (Fig 1B). Thus, pharmacological inhibition of rhomboid protease activity correlates with an enrichment of Hgl and Lgl in lipid rafts in *E*. *histolytica*. Although Lgl has not been surmised to be a EhROM1 substrate, the DCI-induced altered distribution of this subunit may be the result of its covalent connection to Hgl.

### A genetic knockdown of EhROM1 phenocopies DCI-treatment

**Fig 2:**
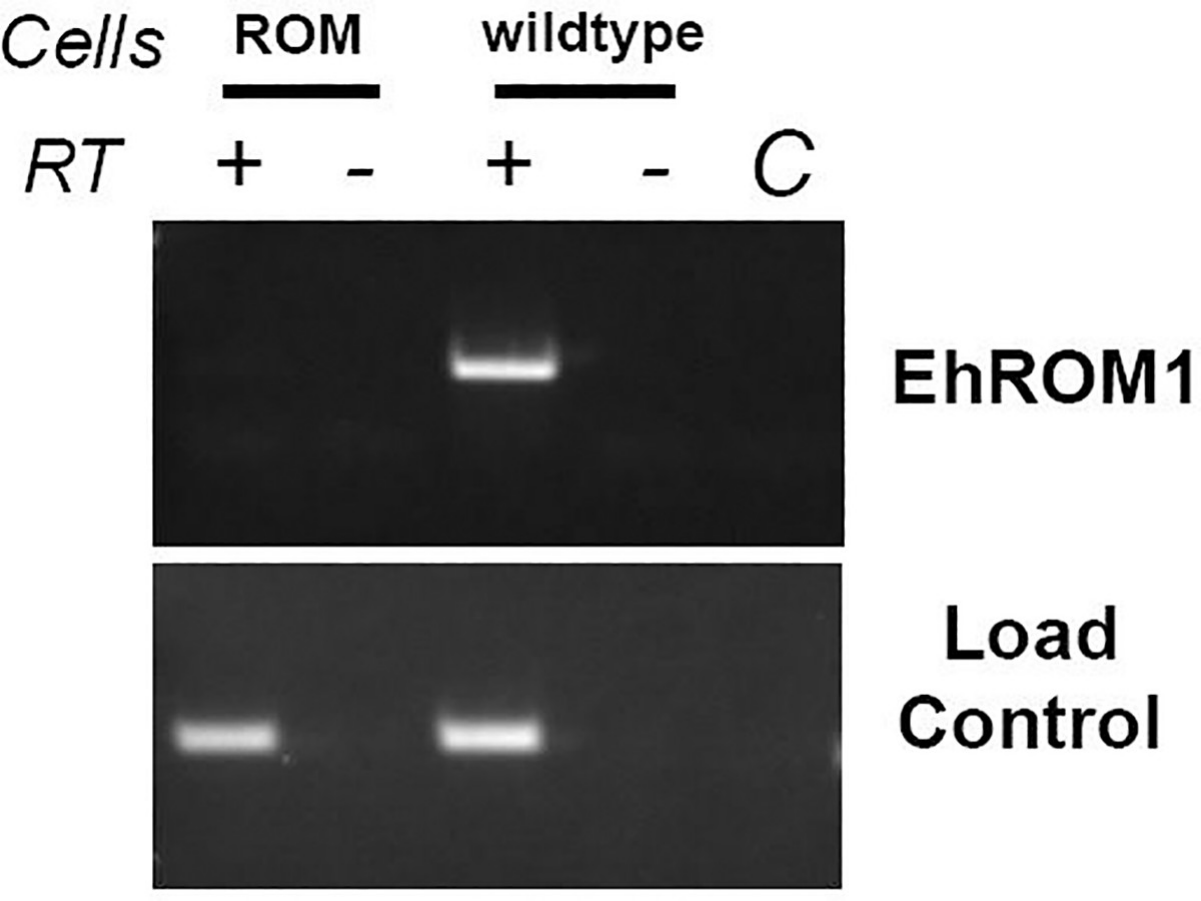
EhROM1 expression is reduced using an RNAi-based approach. RT-PCR was used to detect EhROM1 transcripts in a cell line (ROM) transfected with a RNAi-based Trigger plasmid [33] harboring the EhROM1 gene or in wildtype cells (WT). RT-PCR detection of a small subunit ribosomal RNA (16s-like rRNA; X61116) served as the load control [32]. Reactions, in which the reverse transcriptase step was left out, served as a control for genomic DNA contamination. No genomic DNA contamination was detected (see “RT minus (-)” lanes). Lane C is a control in which no cDNA was added to the PCR reaction. EhROM1 transcript is below the level of detection in the transgenic cell line (ROM).

**Fig 3:**
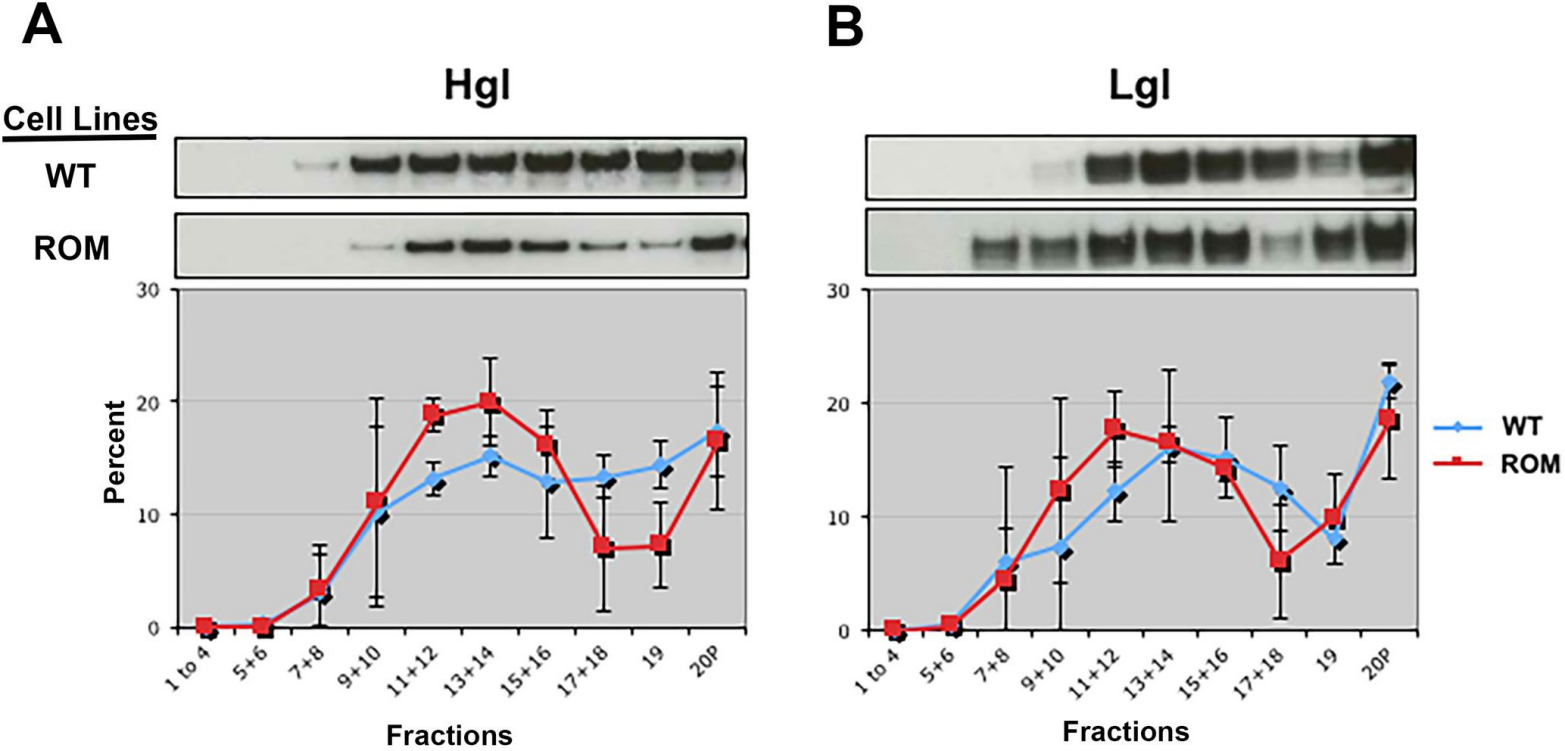
Reduced expression of EhROM1 correlates with enrichment of the heavy (Hgl) and light (Lgl) subunits of the Gal/GalNAc lectins in lipid rafts. Triton-insoluble membranes were isolated from wildtype cells (WT) or T-EhROM1-s (ROM) cells and resolved by sucrose gradient density centrifugation. Nineteen fractions and the non-buoyant pellet (20P) were collected and subjected to western blot analyses using antibodies to the heavy subunit (Hgl) or the light subunit (Lgl). Fractions 1 through 4, and pairs of fractions thereafter, were combined prior to analysis. For each of the subunits, the data presented are the means of scanning densitometry values (n ≥ 2) after conversion to a percentage relative to the values in all other fractions. Representative western blots are provided above each panel. In T-EhROM1-s cells (red), both Hgl and Lgl are enriched in low density lipid rafts (fractions 9–14) when compared to wildtype cells (blue).

DCI shows strong specificity for serine peptidases. Powers and Kam [41] demonstrated that even high concentrations (390–420 μM) of this compound did not inhibit the metalloexopeptidase, leucine aminopeptidase, nor did it inhibit β-lactamase. However, Powers and Kam [41] also demonstrated that DCI could inhibit several esterases, possibly because serine proteases and esterases are similar in their biochemical mechanisms. Since DCI may inhibit non-specific targets, we turned to a genetic approach to corroborate the initial findings. Specifically, we used a cell line (T-EhROM1-s) in which the expression of EhROM1 was reduced using a RNAi-based approach [33]. Knockdown was confirmed by RT-PCR (Fig 2) and this mutant cell line was used for all subsequent experiments. We isolated lipid rafts and characterized the submembrane distribution of the lectin subunits in the mutant cell line. Like DCI-treated cells, T-EhROM1-s cells exhibited augmented levels of Hgl and Lgl in high buoyancy fractions (Fig 3). These data support the notion that there is an inverse relationship between the level of rhomboid protease activity and the level of Hgl and Lgl in lipid raft-like compartments.

### EhROM1 localizes to high-buoyancy triton-insoluble membrane fractions

**Fig 4:**
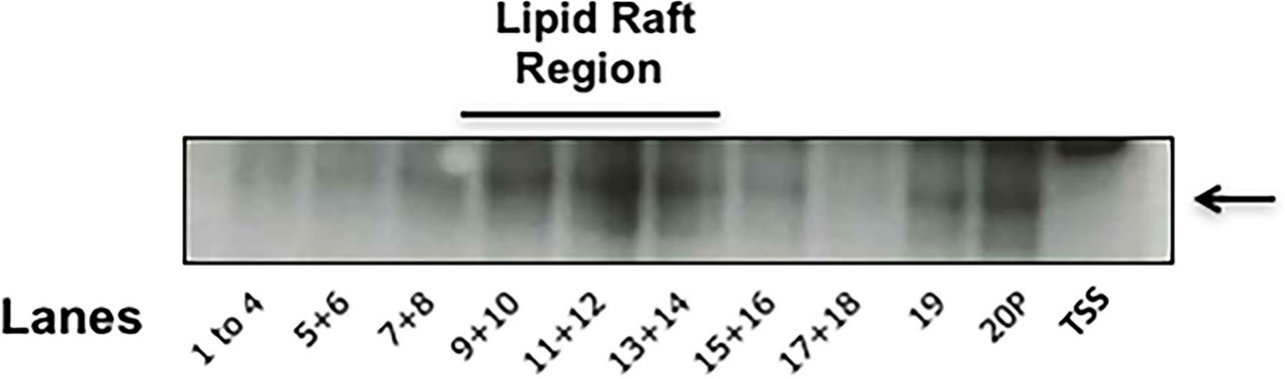
EhROM1 is localized to buoyant triton-insoluble membrane. Triton-insoluble membranes were isolated and further separated by sucrose gradient density centrifugation. Nineteen fractions and the non-buoyant pellet (20P) were collected and subjected to western blot analyses using antibodies specific for EhROM1. TSS represents the triton soluble supernatant. Fractions 1 through 4, and pairs of fractions thereafter, were combined prior to analysis. EhROM1 is enriched in fractions 9 through 14, which have been shown previously to contain raft-like membrane [18]. EhROM1 could not be detected in the TSS.

Since membrane composition may influence the activity of rhomboid proteases [34,35], we tested if EhROM1, itself, was compartmentalized into triton-insoluble membrane by sucrose gradient centrifugation. First, no EhROM1 could be detected in the triton-soluble supernatant by western blot analysis (Fig 4, TSS). This supports the hypothesis that EhROM1 is an intramembrane protease. Second, Western blot analysis of gradient fractions demonstrated that, like Igl, EhROM1 was enriched in highly buoyant fractions reminiscent of rafts (Fig 4). Therefore, like other intramembrane proteases [34,35], EhROM1 may rely on a sterol-rich environment of rafts for its function.

### Cell surface levels of the Gal/GalNAc subunits are higher in T-EhROM1-s cells

We previously showed that enrichment of Gal/GalNAc lectin subunits in rafts correlates with increased galactose-sensitive adhesion [19]. It would follow that T-EhROM1-s cells exhibit increased adhesion to host cells. Contrary to this prediction, it was reported that inhibition of expression or activity of EhROM1 correlates with defects related to reduced adhesion to host cells [32,33]. The purification of rafts by detergent extraction and sucrose gradient centrifugation is carried out with whole cells. Consequently, the protocol does not distinguish between the adhesins in cell surface lipid rafts versus those in intracellular lipid rafts. Despite raft-enrichment, one reason that T-EhROM1-s cells may have impaired adhesion is because less Gal/GalNAc lectin is in cell surface rafts and more Gal/GalNAc lectin is trapped in intracellular rafts. Therefore, we quantified the surface levels of Hgl, Lgl and Igl in T-EhROM1-s cells using biotinylation as previously described [19].

**Fig 5:**
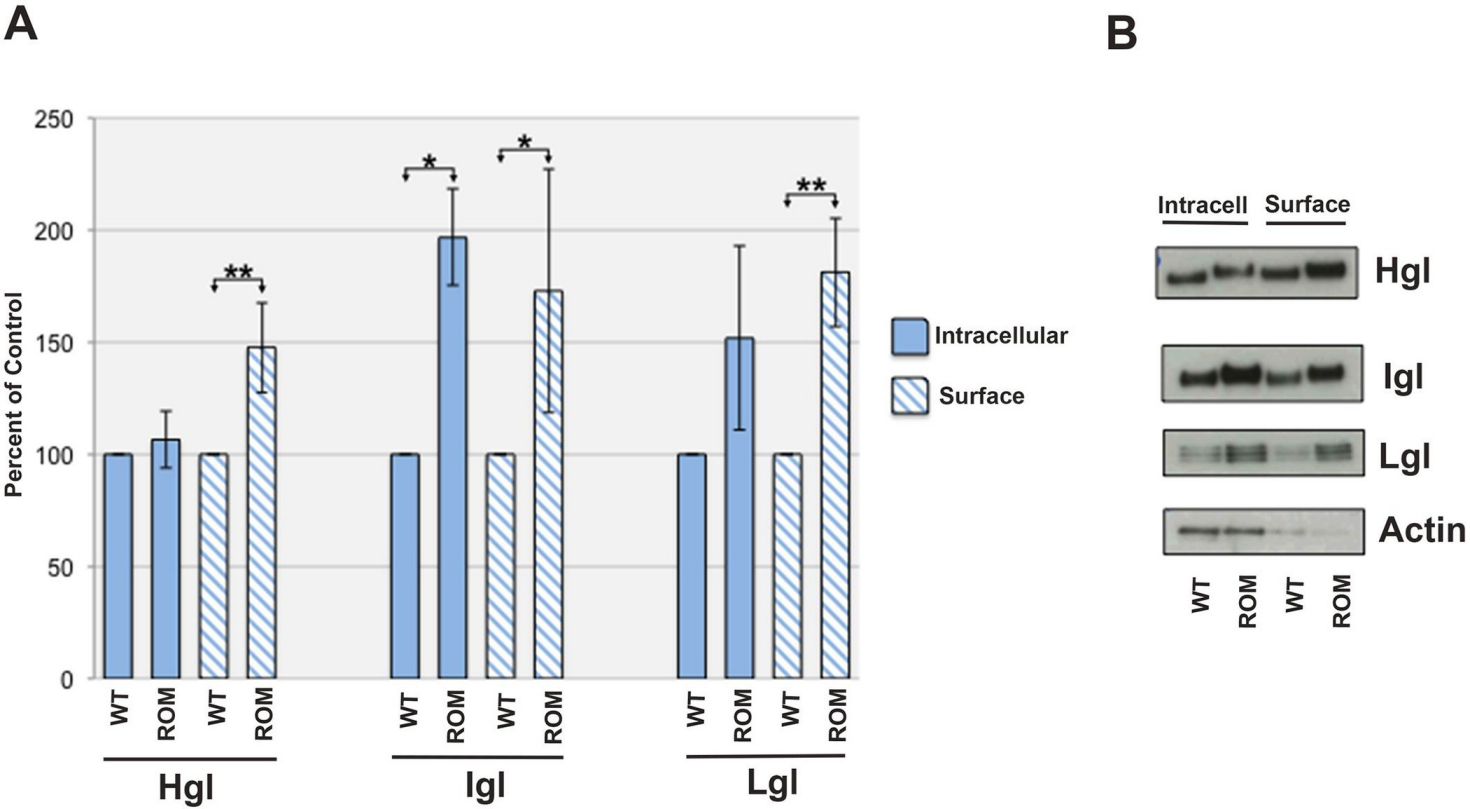
The Gal/GalNAc lectin subunits are enriched on the surface of T-EhROM1-s cells. Wildtype (WT) and T-EhROM1-s (ROM) cells were surface biotinylated. Labeled surface proteins were captured by avidin affinity chromatography. Both purified biotinylated surface proteins (hashed bars) and the unbound flow-through fraction, which represents intracellular protein (solid bars), were analyzed by SDS-PAGE and western blotting with antibodies specific for the heavy subunit (Hgl), the intermediate subunit (Igl) or the light subunit (Lgl). (A) Mean values of scanning densitometry data (n ≥ 3), reported as a percentage of total Hgl, Igl, or Lgl in WT cells, which was arbitrarily set to 100%. There is a statistically significant increase in the level of all three subunits on the surface of T-EhROM1-s amoebae (*P<0.05; **P<0.01) (hashed bars). There are also increases in the intracellular levels of Igl, Lgl, and Hgl as shown by analysis of the intracellular protein (solid bars). Only the increase in the intracellular level of Igl is statistically significant (*P<0.05). (B) Representative western blots of intracellular (intracell) and surface proteins showing an increase in the level of Hgl, Igl and Lgl on the surface of mutant cells compared to that of wildtype cells. Actin, a cytosolic protein, serves as a control for premature cell breakage.

Whole cells were exposed to sulfo-NHS-SS-biotin to label surface proteins. Cell lysates were subjected to avidin-agarose affinity chromatography. Equivalent fractions of the starting cell lysate and affinity purified protein were analyzed for the lectin subunits by western blotting. Surprisingly, surface biotinylation and affinity chromatography revealed that there were significantly higher levels of all three subunits on the surface of mutant cells compared to the surface of control cells (Fig 5A (hashed bars), Fig 5B). Using immunofluorescence microscopy with α-Hgl antibody, Baxt *et al*. [32] concluded that there was no increase in the surface level of Hgl when EhROM1 was silenced. The incongruence of our data with that of Baxt *et al*. [32] is likely a reflection of the different techniques used to quantify cell-surface proteins. We also observed an increase in the level of *intracellular* lectin subunits (Fig 5A (solid bars), Fig 5B). These increases were statistically significant for Igl (*P<0.05), but not for Hgl and Lgl. These data are consistent with that of Baxt *et al*., [32], who reported an increase, albeit not statistically significant, in the level of Hgl in mutant cells as detected by the ELISA method. Together, with the increases seen for the cell surface-localized subunits in this study, these observations suggest that there may be more Hgl, Lgl, and Igl, overall in the mutant cell line.

### Hgl and Igl exhibit a higher molecular weight in T-EhROM1-s cells

**Fig 6:**
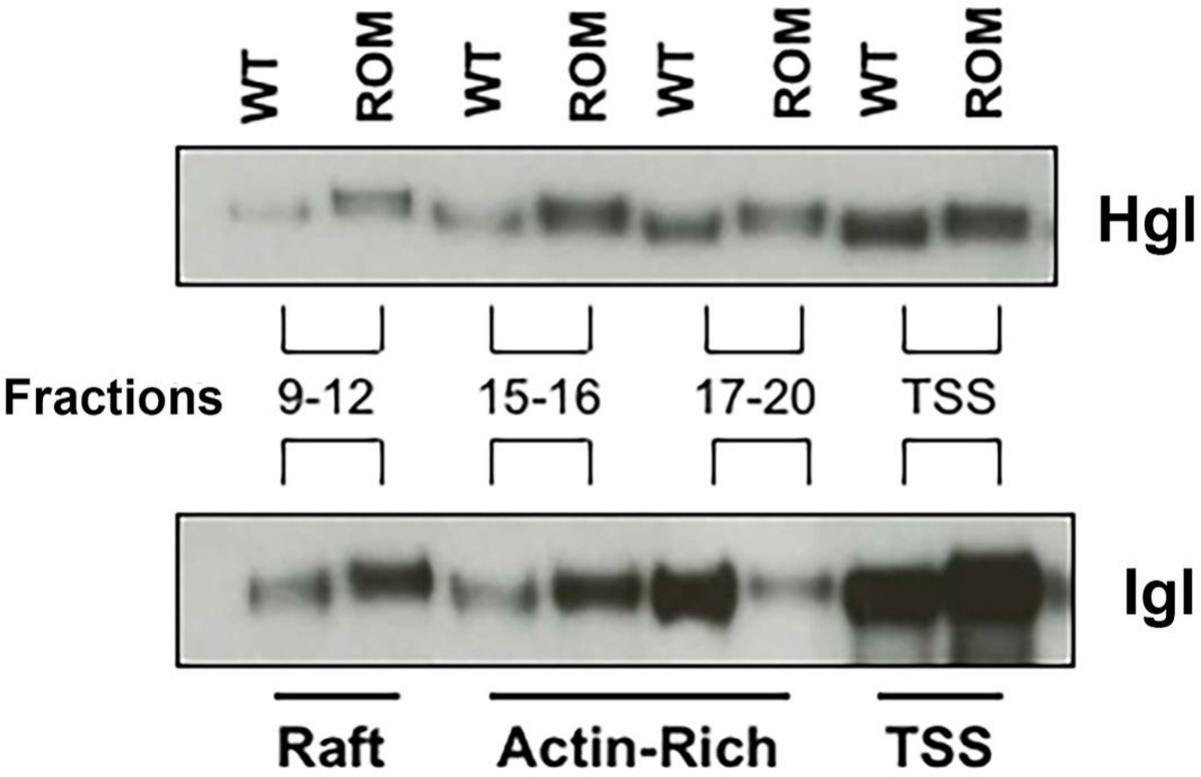
Hgl and Igl exhibit a higher molecular weight in T-EhROM1-s cells. Triton-insoluble membranes were isolated from wildtype (WT) or T-EhROM1-s (ROM) cells and resolved by sucrose gradient density centrifugation. Nineteen fractions were collected. Fractions 9 through 12 (lipid rafts), 15 and 16 (actin-rich membrane), or 17 through 19 (actin-rich membrane) were each combined and subjected to SDS-PAGE in a MOPS buffer system, which is superior in its ability to resolve high molecular weight proteins. Following SDS-PAGE, western blot analysis was carried out using antibodies specific for the heavy subunit (Hgl), or the intermediate subunit (Igl). The triton-soluble supernatant (TSS) was also analyzed in the same manner. Hgl and Igl exhibit a higher molecular weight in all membrane types in the mutant cell line.

It was intriguing that the T-EhROM1-s cell line exhibited enrichment of the Hgl and Lgl in rafts, and higher levels of the Gal/GalNAc lectin subunits at the surface, but was impaired in many adhesion-related functions [33]. Since Hgl is a putative substrate for EhROM1 [31], perhaps proper cleavage of Hgl, by EhROM1 is required for suitable function of the Gal/GalNAc lectin. To test this, we isolated lipid rafts and examined the size of the lectin subunits in both mutant and control cells using SDS-PAGE and western blotting in a MOPS buffer system, which provides superior separation of high molecular weight species. In the mutant cell line, Hgl was larger than its counterpart in control cells (Fig 6). This was true for this subunit in all membrane domains (i.e., rafts, actin-rich membrane, and detergent-sensitive membrane) (Fig 6). These data support the supposition that Hgl is a substrate of EhROM1 [31].

We were surprised to see that the size of Igl was also increased in all membrane domains in the mutant cell line (Fig 6). These observations suggest that Igl is also a substrate of this protease. There is evidence that rhomboid proteases can act on hydrophilic sequences outside of transmembrane domains [42–44]. Thus, it is conceivable that GPI-linked Igl could be a substrate of EhROM1 even though it is predicted to reside completely outside of the membrane.

### Assembly of the Gal/GalNAc lectin trimer is not inhibited in T-EhROM1-s cells

**Fig 7:**
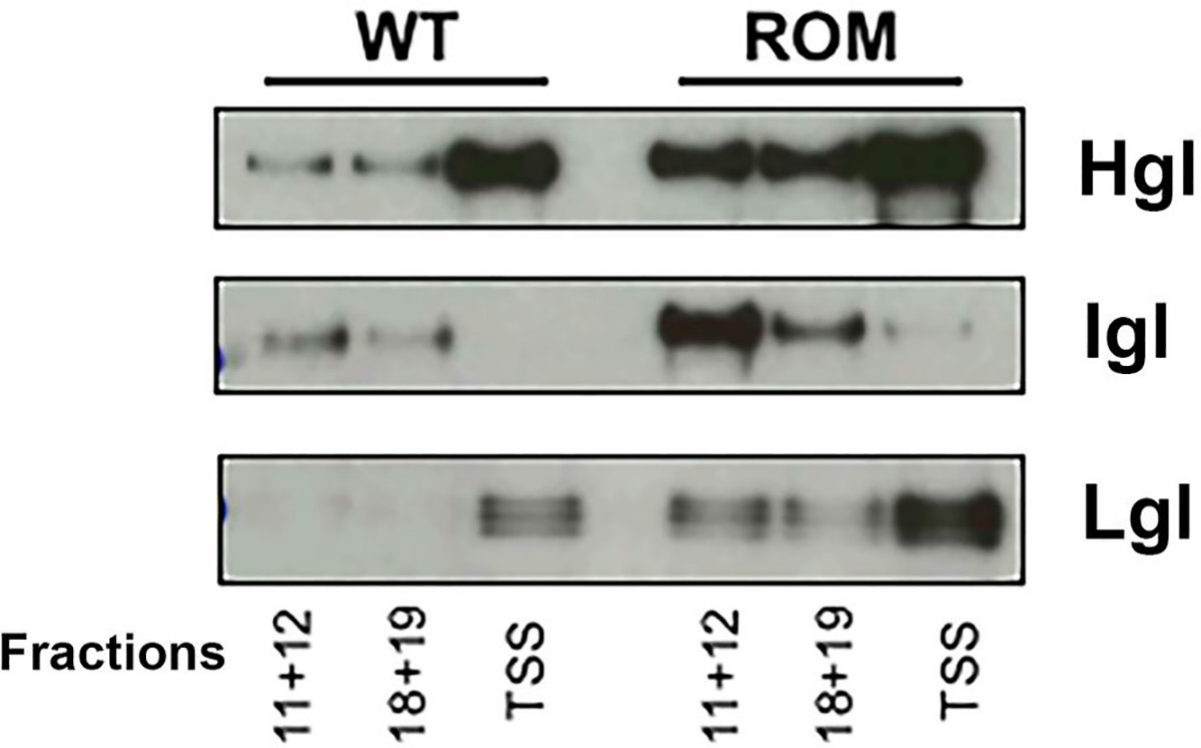
Assembly of the Gal/GalNAc lectin trimer is not inhibited in T-EhROM1-s cells. Triton-insoluble membranes were isolated from wildtype (WT) or T-EhROM1-s (ROM) cells and resolved by sucrose gradient density centrifugation. Nineteen fractions were collected. Fractions 11 and 12 (lipid rafts), or 18 and 19 (actin-rich membrane), were each combined and subjected to immunoprecipitation using a monoclonal α-Hgl antibody. The triton-soluble supernatant (TSS) was also analyzed in the same manner. These narrow ranges of fractions were chosen to increase the probability that no membrane type (e.g., lipid rafts) would be contaminated with any other membrane type (e.g. actin-rich membrane). Precipitated proteins were resolved by SDS-PAGE and assessed by western blotting using antibodies specific for the heavy subunit (Hgl) (polyclonal), the intermediate subunit (Igl) or the light subunit (Lgl). Pull down of Hgl reveals co-association with Lgl in all membrane types. In T-EhROM1-s cells, more Lgl associates with Hgl in the lipid rafts (11+12) because both subunits are enriched in rafts in that cell line. Little Igl co-associates with Hgl in the TSS, because the majority of Igl is constitutively localized to buoyant triton-insoluble raft-like membrane.

The shift in size of Hgl and Igl in the T-EhROM1-s cell line raised the possibility that the subunits are not interacting with each other normally. This, in turn, could explain the adhesion defect in the mutant cell line. To test this, we examined interaction among the subunits using an immunoprecipitation approach. Detergent-sensitive membrane and various fractions of detergent-resistant membrane, resolved by sucrose gradient centrifugation, were mixed with α-Hgl monoclonal antibody. Following magnetic purification, the immune complexes were resolved by SDS-PAGE and analyzed by western blotting using α-Hgl, α-Lgl, or α-Igl antibodies. In all membrane types in the mutant cells, there was no alteration in the interaction profiles of the Gal/GalNAc lectin subunits (Fig 7). Therefore, the increased size of Hgl and Igl in the T-EhROM1-s cell line did not seem to hinder the assembly of trimers, and thus, cannot explain the adhesion defect. Perhaps the aberrant size of the subunits masks crucial binding sites and/or alters the three-dimensional structure of the trimer, in a manner that inhibits its function.

## Discussion

The principal finding of this study is that rhomboid protease activity may directly or indirectly regulate the subunit size and the submembrane location of the Gal/GalNAc lectin in *E*. *histolytica*. Pharmacological treatment of cells with a known rhomboid protease inhibitor, DCI, resulted in an enrichment of Hgl and Lgl in rafts. The interesting phenotype observed after DCI-treatment provided the impetus to focus on a genetic knockdown of EhROM1 expression (T-EhROM1-s) for the remainder of the study. Like DCI-treated cells, T-EhROM1-s cells had augmented levels of Hgl and Lgl in lipid rafts. In the mutant cell line, there was also a significant increase in the level of all three Gal/GalNAc subunits on the cell surface and an increase in the molecular weight of two of the subunits, Hgl and Igl. Finally, it was observed that EhROM1, itself, is localized to buoyant triton-insoluble membranes.

In previous studies, enrichment of Hgl and Lgl in lipid rafts was correlated with an increase in galactose-sensitive adhesion to host components [19]. However, in the current study, we observed enrichment of Hgl and Lgl in lipid rafts in a cell line known to display defects related to adhesion-related processes [33]. Thus, for the first time, we show that enrichment of the Hgl-Lgl dimer in lipid rafts, and trimer formation, is not sufficient to enhance the adhesive capacity of the parasite. One possible explanation for the accumulation of Hgl and Lgl in rafts in the mutant cell line is that cleavage of the Gal/GalNAc lectin subunits by EhROM1 is necessary for recycling or exit of these subunits out of rafts. This would be consistent with previous reports that demonstrate that rhomboids play a role in dismantling adhesion complexes in a variety of parasites (reviewed in [21]) including *Plasmodium* [27,28,45,46], *Toxoplasma* [22], and *Trichomonas* [30]. Here, we also see, for the first time, an enrichment of the Hgl-Lgl dimer in lipid rafts in the absence of extracellular ligand binding. Overall, this insinuates a model in which the dimer is constantly entering and exiting rafts, with the majority of Hgl-Lgl outside of rafts at steady-state. If egress from rafts directly or indirectly relies on rhomboid protease activity, then a lack of rhomboid activity would naturally alter this balance resulting in an accumulation of the dimer in these microdomains.

That the lectin subunits require EhROM1 activity to exit rafts would also be consistent with a hypothesis put forth by Baxt *et al*. [31]. Specifically, these authors demonstrated that EhROM1 was localized to caps, also known as uroids, which are structures that are rich in Gal/GalNAc lectin and found at the trailing edge of motile cells. Uroids form during motility for the purpose of concentrating and shedding bound host proteins, such as antibodies. Based on these findings, Baxt *et al*. [31] hypothesized that EhROM1 regulates the release of individual lectin subunits, and their bound host proteins, from uroids. This hypothesis is supported by the current study, which shows that EhROM1 is found in triton-insoluble membrane with the Hgl-Lgl dimer and that this dimer accumulates in rafts in cells with reduced EhROM1 expression. Interestingly, our previous study showed that uroids are, themselves, rich in rafts [47]. Thus, this structure may, indeed, be a compartment in which EhROM1 can act on the Gal/GalNAc lectin.

Riestra *et al*. [30] mutated a putative rhomboid cleavage site of a cell surface-localized substrate of *T*. *vaginalis* ROM (TvROM), which rendered that substrate unavailable for cleavage. Expression of this mutated substrate resulted in its accumulation on the cell surface and increased *T*. *vaginalis*-host adhesion. We also observed a significant increase in the level of Gal/GalNAc lectin subunits on the surface of the cell line with reduced EhROM1 expression. Remarkably though, this increase was *not* correlated with enhanced adhesion. In fact, lower EhROM1 expression is correlated with reduced adhesion [32,33]. Perhaps the adhesion-related defects in the mutant cell line are related to the aberrant size of Hgl and Igl. Immunoprecipitation assays demonstrated that the larger Gal/GalNAc subunits were able to interact with each other in a normal fashion; but, complexes possessing these larger subunits may no longer be able to interact with host ligands and/or other *E*. *histolytica* proteins necessary for adhesion. For instance, crucial binding sites may be hidden and/or the three-dimensional structure may be anomalous in un-cleaved lectin subunits.

The increased molecular weight of Igl in the mutant cell line was particularly intriguing. It suggests that Igl may be a substrate of EhROM1, even though this subunit is surmised to be GPI-anchored and localized completely outside the membrane. More recently it has been shown that some rhomboid proteases can cleave sequences within hydrophilic sequences outside of transmembrane domains [42–44]. In these cases, the hydrophilic peptide substrates bend into the protease active site from above the membrane plane (reviewed in [48]). Our observations suggest the EhROM1 may also be capable of cleaving sequences outside of the membrane environment. Given recent evidence that rhomboid proteases rely on substrate concentration [49] and the lipid environment [50,51], more than consensus sequences, to interact with substrates, additional direct tests of cleavage will be necessary to conclude that Igl is an authentic substrate of EhROM1.

The mechanism by which EhROM1 recognizes its substrates remains to be determined. Previously, it was established that EhROM1 displays atypical substrate specificity. Along with *Trichomonas vaginalis* ROM1 (TvROM1) [30] and *Plasmodium falciparum* Rhomboid 4 (PfROM4) [27], EhROM1 is one of three parasite rhomboid proteases that cannot process Spitz, a substrate from *Drosophila* that is cleaved by most known rhomboid proteases [31]. A variety of processes for rhomboid-substrate recognition seem to exist [48]. For instance, cleavage of thrombomodulin by vertebrate rhomboid-2, RHBDL2, is directed by the substrate’s cytoplasmic tail rather than by sequences in its transmembrane region [52]. Furthermore, a recent study of the activity of ten diverse rhomboid proteases, in an *in vitro* reconstituted membrane system, demonstrates that the requirement for specific sequences in substrates is much less stringent than previously thought and instead hydrolysis is likely driven, in part, by substrate concentration (rate-driven) [49]. Thus, co-localization of Hgl or Igl with EhROM1, in buoyant triton-insoluble membrane, may be sufficient for their proteolysis by EhROM1. In fact, the environment of the lipid raft, itself, may contribute to the ability of EhROM1 to target Hgl and Igl. Two previous studies [50,51] demonstrated that it was possible to transmute non-substrates into substrates simply by changing membrane composition with membrane-disrupting agents, including the cholesterol-binding agent, methyl-β-cyclodextrin.

Although Baxt *et al*. showed that Hgl was cleavable by EhROM1 when co-expressed in a mammalian cell system [31], this has never been demonstrated directly in *E*. *histolytica* cells. Therefore, we cannot rule out the possibility that the observed changes in the mutant cell line are indirect. For example, reduced cleavage of another unknown EhROM1 substrate indirectly leads to the accumulation of the Hgl-Lgl dimer in rafts and/or to the change in size of Hgl and Igl. In Archaea, a link between rhomboid protease activity and protein glycosylation has been proposed [53]. Perhaps a similar link exists in eukaryotes and if so, changes in the glycosylation patterns of Hgl and Igl may also account for their size difference in T-EhROM1-s cells. Nevertheless, the study provides significant insight into parasite-host adhesion for *E*. *histolytica*. Given the importance of adhesion for virulence of *E*. *histolytica*, new information on the molecular mechanisms governing host colonization may contribute to the development of novel drugs for this devastating global disease.

## Supporting information

S1 Fig (PDF)

S2 Fig (PDF)

S3 Fig (PDF)

S4 Fig (PDF)

S5 Fig (PDF)

S6 Fig (PDF)

S7 Fig (PDF)
