# Supplementary figures and images for: Reduced expression of a rhomboid protease, EhROM1, correlates with changes in the submembrane distribution and size of the Gal/GalNAc lectin subunits in the human protozoan parasite, Entamoeba histolytica
Source: PLoS One. 2020 Mar 5;15(3):e0219870. doi: 10.1371/journal.pone.0219870 (PMC7058331; doi:10.1371/journal.pone.0219870)

Fig 1

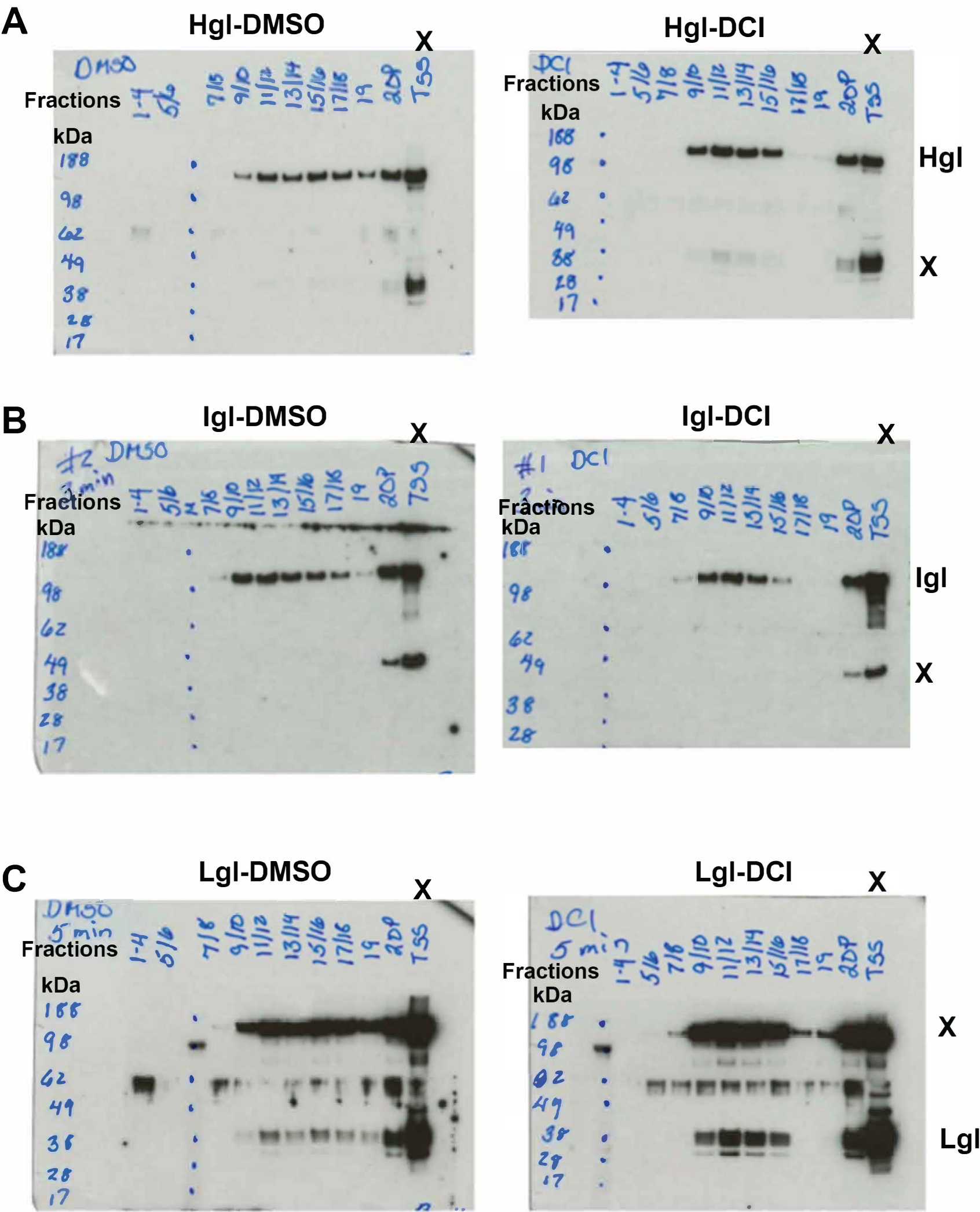

Supplement: S1 Fig — (PDF) [file pone.0219870.s001.pdf]

Fig 2

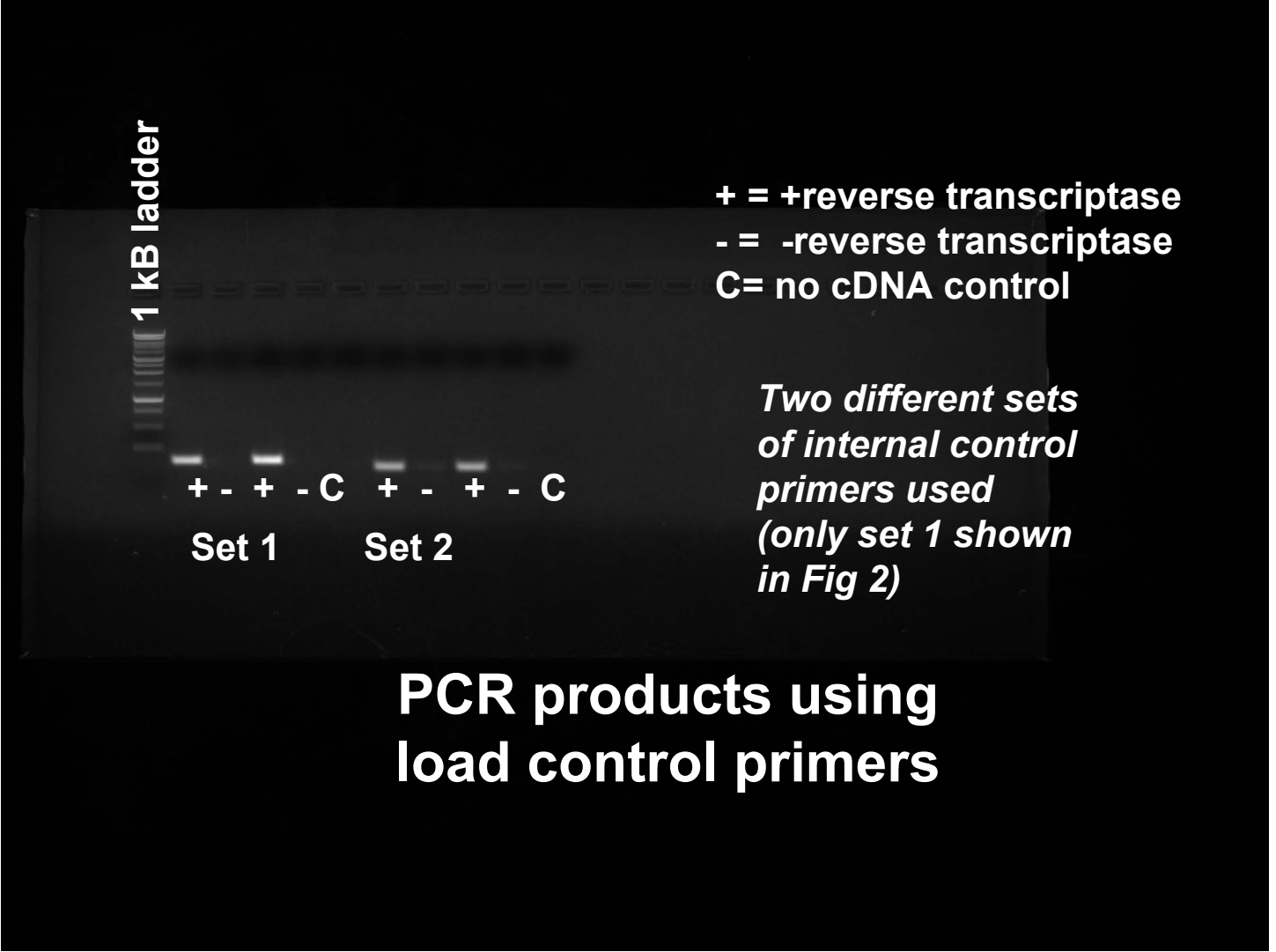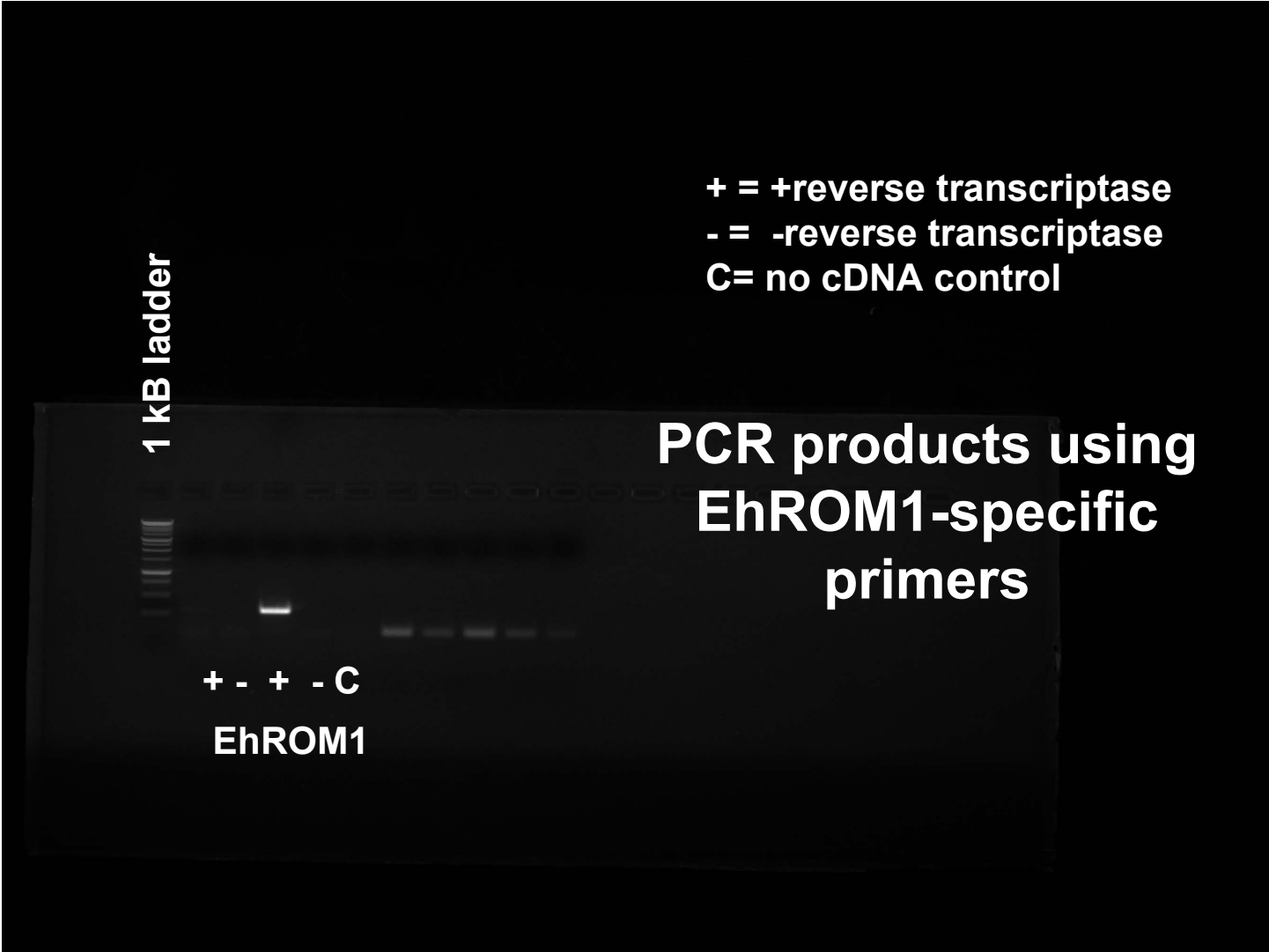

Supplement: S2 Fig — (PDF) [file pone.0219870.s002.pdf]

Fig 3

A

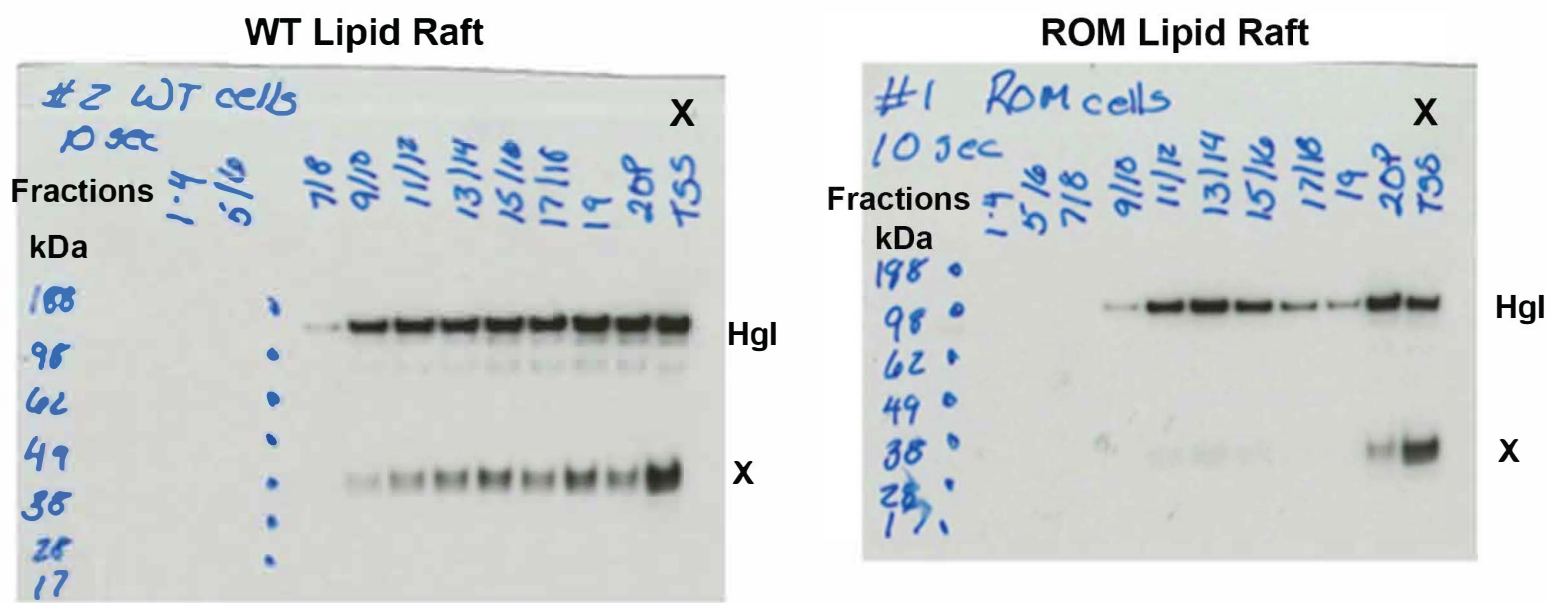

B

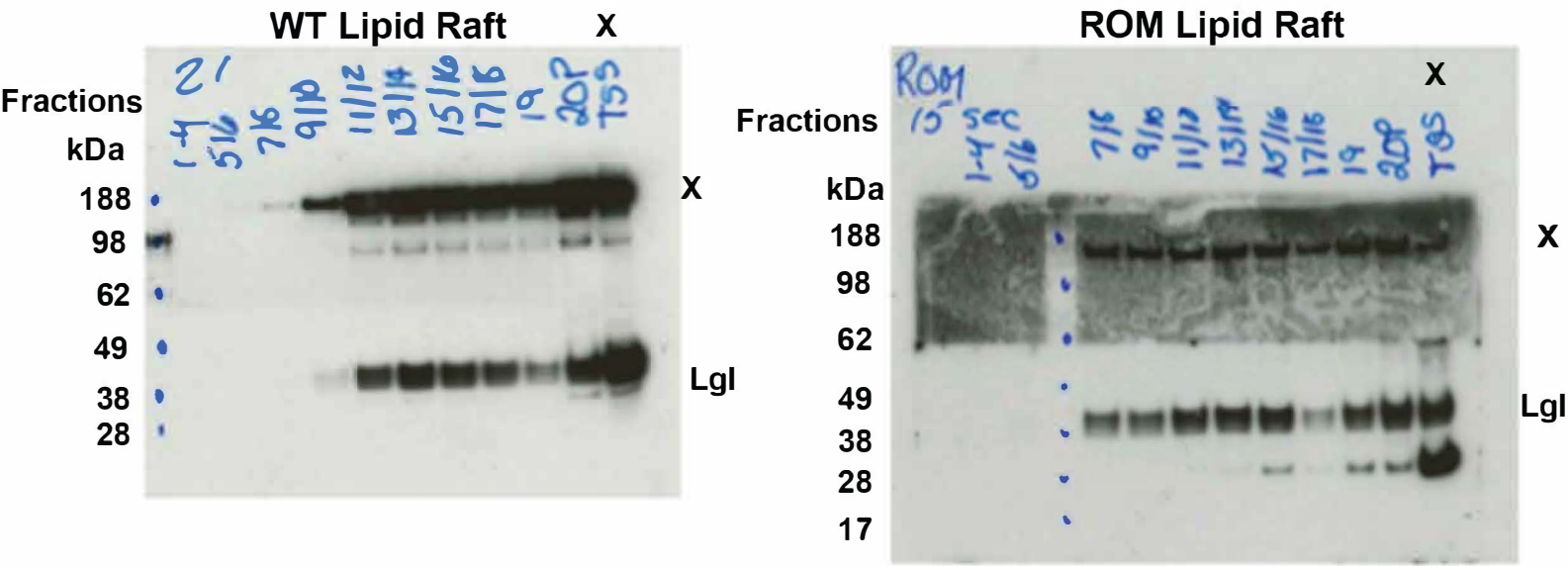

Supplement: S3 Fig — (PDF) [file pone.0219870.s003.pdf]

Fig 4

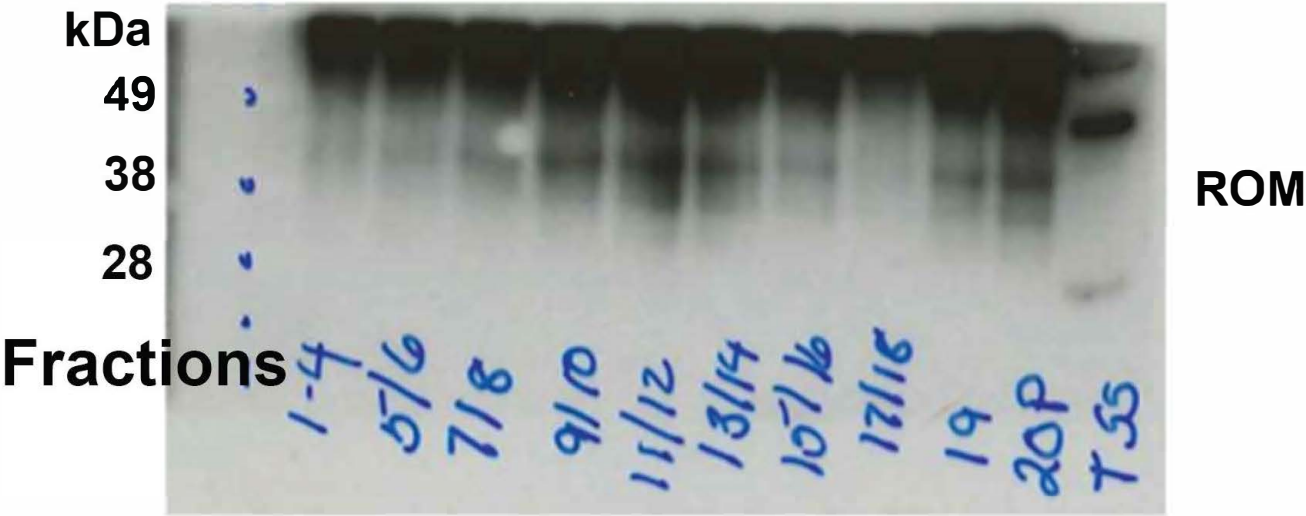

Supplement: S4 Fig — (PDF) [file pone.0219870.s004.pdf]

Fig 5

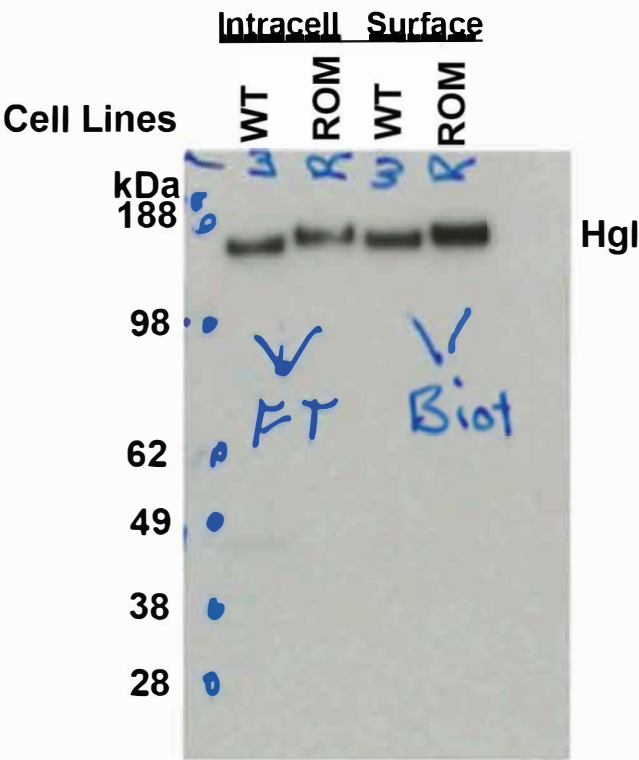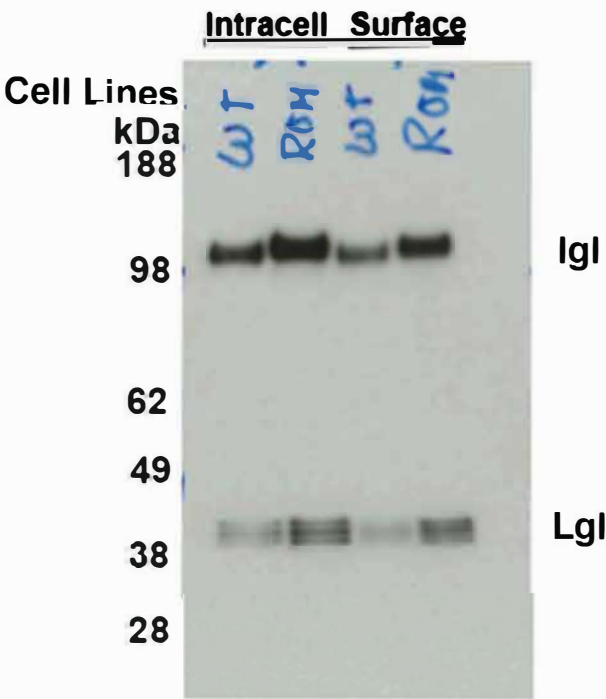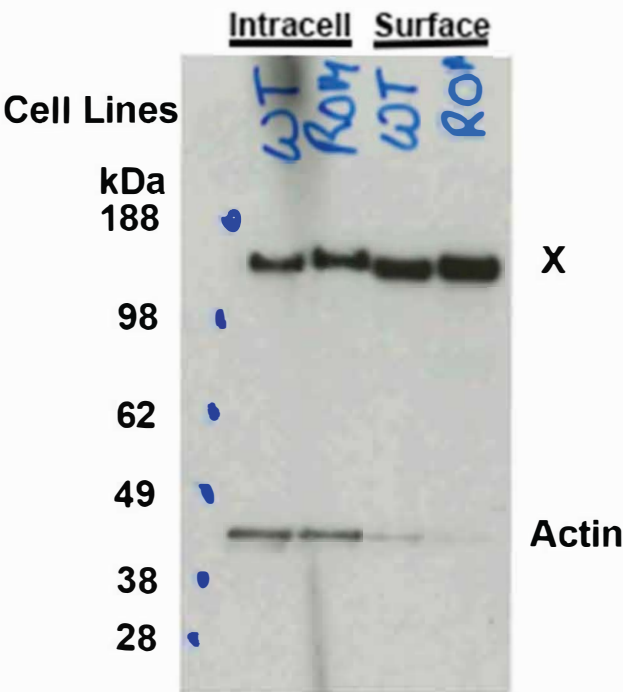

Supplement: S5 Fig — (PDF) [file pone.0219870.s005.pdf]

Fig 6

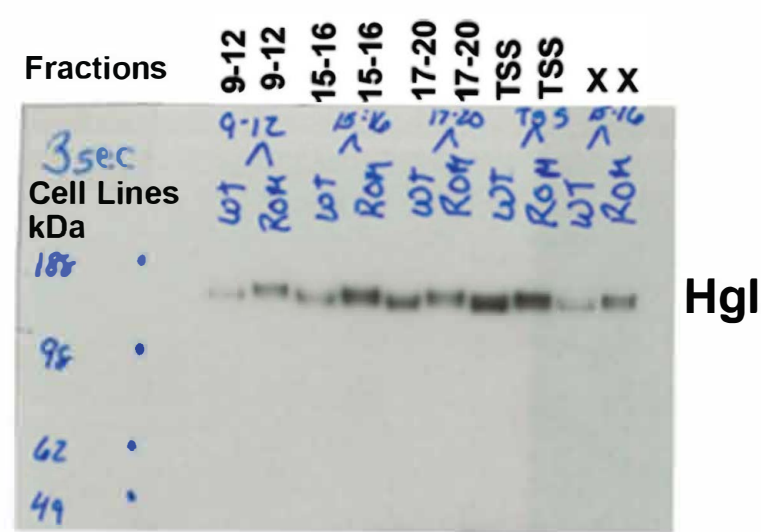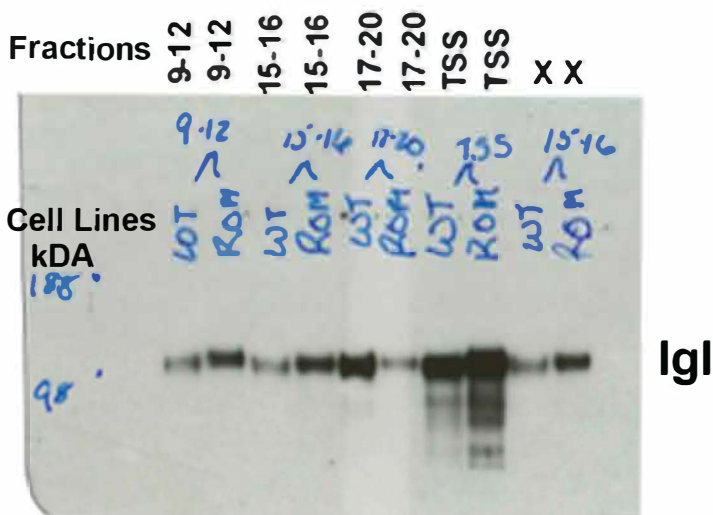

Supplement: S6 Fig — (PDF) [file pone.0219870.s006.pdf]

Fig 7

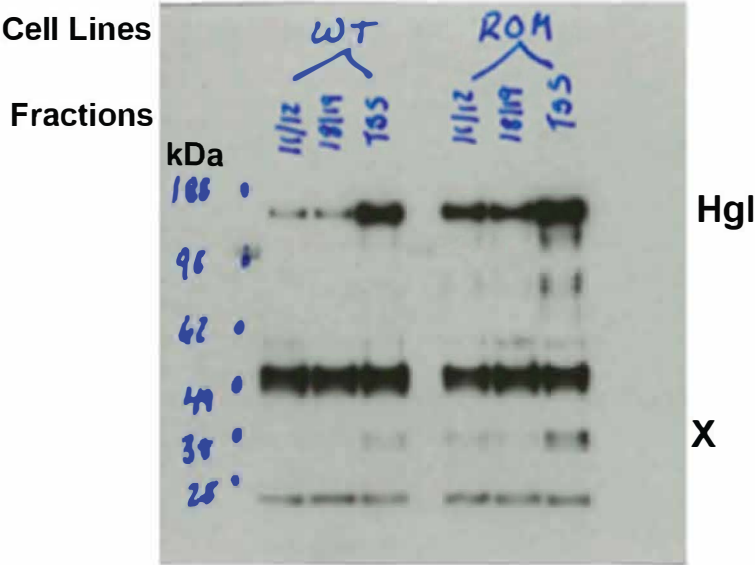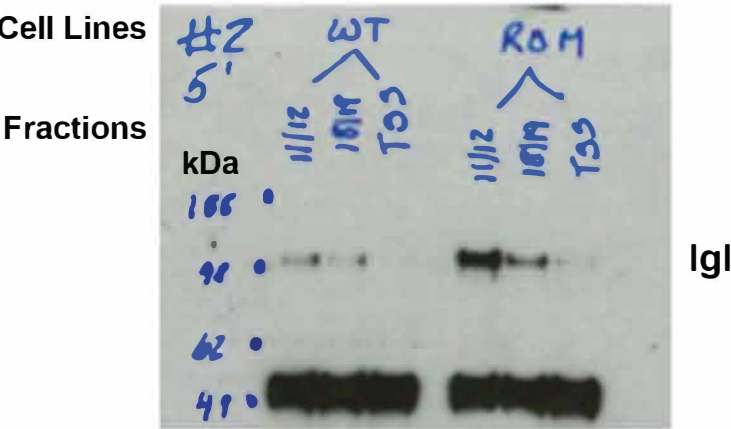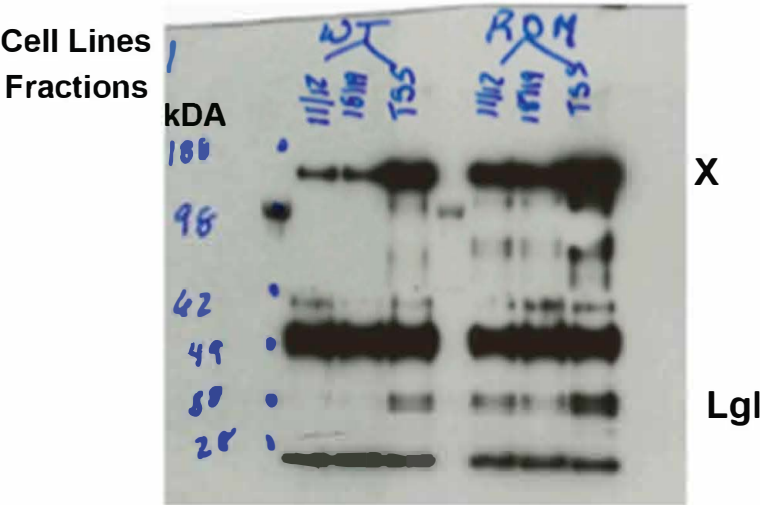

Supplement: S7 Fig — (PDF) [file pone.0219870.s007.pdf]
